# 2, 8 Dihydroxyadenine urolithiasis: A case report and review of literature

**DOI:** 10.4103/0971-4065.50680

**Published:** 2009-01

**Authors:** P. Sreejith, K. L. Narasimhan, V. Sakhuja

**Affiliations:** Department of Nephrology, PGIMER, Chandigarh, India; 1 Department of Paediatric Surgery, PGIMER, Chandigarh, India

**Keywords:** Adenine phosphoribosyl transferase deficiency, urolithiasis, 2, 8 dihydroxyadenine

## Abstract

Adenine phosphoribosyl transferase deficiency is a rare metabolic abnormality presenting with 2,8 dihydroxyadenine urolithiasis. The stones are characteristically radiolucent and therefore need to be differentiated from uric acid stones which are also radiolucent and have identical chemical reactivity. No cases of 2, 8- dihydroxyadenine urolithiasis have been reported from India. We report a 3 year old child with 2, 8- dihydroxyadenine urolithiasis and acute renal failure.

## Introduction

2, 8-dihydroxyadeninuria is a rare, inherited cause of urolithiasis. To the best of our knowledge, no case of 2,8-dihydroadenine urolithiasis has been reported in Indian literature. We report here the case of a three year-old child with 2, 8-dihydroxyadenine urolithiasis and renal failure.

## Case Report

**Figure 1:**
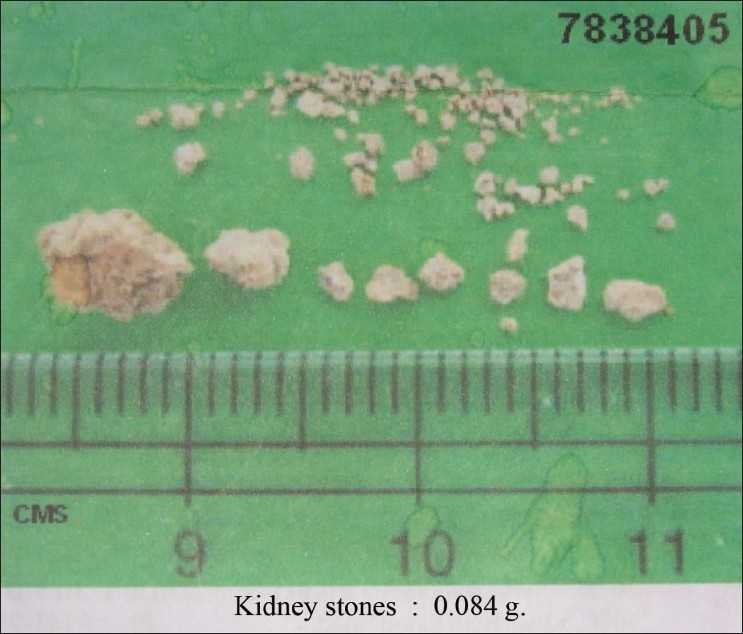
The 2,8 dihydroxyadenine stones removed from our patient on ureterolithotomy

A three year-old girl presented with a history of vomiting and not passing urine for 24 hours. She was apparently healthy earlier and gave no history of fever, crying during micturition, hematuria, or graveluria. There was also no history of renal stone disease in the family. Physical examination revealed facial puffiness and tachypnea. She was afebrile and her blood pressure was 100/60 mm Hg. The systemic examination did not reveal any abnormality. Investigations revealed anemia (Hb 8.2 g/dL), pyuria, renal failure (blood urea 90 mg/dL, serum creatinine 3.7 mg/dL) with hyperkalemia (K^+^ 6.6 mEq/L), and severe metabolic acidosis (pH 7.27, pO_2_ 66 mm Hg, pCO_2_ 21 mm Hg, HCO3- 10 mEq/L, BE -15) for which she received a session of peritoneal dialysis. An ultrasonography of the abdomen revealed calculi in the right lower calyx and at the lower end of the right ureter. The left kidney was normal in size and shape and did not show any calculi or hydronephrosis. A plain X-ray KUB did not show any radio-opaque shadow, indicating that the calculi were radiolucent. A DJ stent was introduced into the right ureter after which her urine output and renal function improved (serum creatinine 1.2 mg/dL). A cystoscopic ureterolithotomy was subsequently performed on the right side. The calculi were found to be soft and friable with an irregular surface [Figure 1], and they were removed piecemeal. The stone was sent for analysis by Fourier transform infrared spectroscopy which showed that it was composed entirely of 2, 8-dihydroxyadenine. The patient was started on allopurinol (10 mg/kg/day) and has been asymptomatic with normal renal function for the last four months, albeit with a small residual calculus in the right lower calyx.

## Discussion

2, 8-Dihydroxyadenine urolithiasis is the result of a metabolic abnormality due to the deficiency of the enzyme, adenine phosphoribosyl transferase (APRT), a salvage enzyme present in all human cells.[1] It catalyzes the formation of adenine monophosphate from adenine in the presence of phosphoribosyl pyrophosphate. In the absence of APRT, adenine is metabolized by xanthine oxidase to 2, 8-dihydroxyadenine via the intermediate, 8-hydroxyadenine. 2,8-Dihydroxyadenine is excreted by the kidneys and is insoluble in urine at any range of physiological pH, resulting in 2, 8- dihydroxyadenine crystalluria and urolithiasis.

APRT deficiency is inherited in an autosomal recessive manner and the gene is located on chromosome 16q24. Twenty-four functionally significant APRT mutations have been identified. Based on the level of residual enzyme activity in erythrocyte lysates, two types of APRT deficiency have been described. In Type I APRT deficiency, enzyme activity is virtually absent whereas in Type II APRT deficiency, enzyme activity ranges from 10 to 25% in erythrocyte lysates. Type I deficiency is seen in the Caucasian population whereas Type II deficiency is almost exclusively seen in the Japanese. The frequency of heterozygosity for APRT deficiency is 0.4–1.1% in Caucasians and 0.5–1.2% in the Japanese.[2]

**Figure 2:**
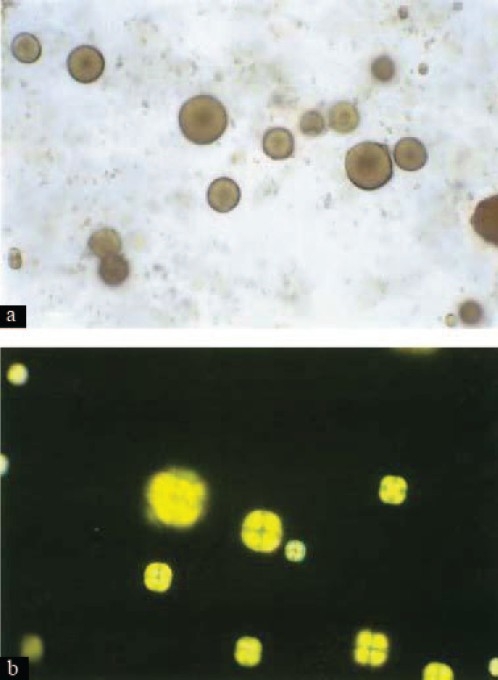
(a) Light microscopy of urine sediment - round, reddish-brown, 2,8-DHA crystals with dark outlines and central spicules, (b) Polarized light microscopy of urine sediment - characteristic 2,8-DHA crystals with the central Maltese cross pattern. (Original magnification × 400.) Taken from: Edvardsson V, et al. Am J Kidney Dis. 2001 Sep;38(3):473-80

Urolithiasis is the most common manifestation of APRT deficiency,[2] with patients presenting with urinary tract infection, macroscopic hematuria, and colic or urinary retention. The stones are characteristically radiolucent and therefore, need to be differentiated from uric acid stones which are also radiolucent and have identical chemical reactivity.[3] 2, 8-dihydroxyadenine stones are soft and friable with an irregular surface whereas urate stones are hard and difficult to break, have a smooth surface, and have faint yellow color. The diagnosis is usually made after detailed analysis of the stones using infrared spectroscopy, with confirmation by assay of APRT enzyme activity in erythrocytes. Urine sediment in these patients shows the characteristic round, reddish-brown 2,8-dihydroxyadenine crystals with dark outlines and central spicules. Polarized light microscopy of the urine sediment shows 2, 8-dihydroxyadenine crystals with a central maltese-cross pattern [Figure 2].

Individuals with APRT deficiency may also present with acute renal failure due to crystalluria or obstructive uropathy and chronic kidney disease in the absence of urolithiasis,[2] which is thought to be the result of microcrystalline-induced nephropathy.

Another characteristic presentation is in the form of recurrent disease after kidney transplantation.[4,5] This occurs in instances when the disease was not recognized prior to the transplantation with recurrence in the graft leading to graft dysfunction. Hypoplasia of the kidneys and other developmental abnormalities of the urogenital tract have also been documented in patients with APRT deficiency.[2] Even though this is an inherited disease, it does not always manifest in childhood, with 60–75% of the patients presenting in adulthood, the median age at diagnosis being 37 years.

Approximately 8–21% of individuals with APRT deficiency are asymptomatic and are identified on screening of their family members.[2] Varying ability to supersaturate the urine and differences in dietary practices may explain the existence of affected and asymptomatic siblings in several families. APRT deficiency is a rarely recognized disease, with the majority of cases being reported from Japan, France,[3] and Iceland.[6] The largest number of cases has been reported from Japan; among the 200 individuals identified in Japan, 45% had type I defect and 8–21% were asymptomatic.[2]

2, 8-dihydroxyadenine formation can be easily controlled with allopurinol, which is administered in a dose of 300 mg/day in adults (10 mg/kg/day in children) in the absence of renal failure. After treatment with allopurinol, adenine becomes the predominant purine excreted in urine instead of 2, 8-dihydroxyadenine; a high fluid intake is also advised.
